# Endovascular coil embolization compared to surgical ligation of the uterine artery in a non-human primate model in a model of preeclampsia

**DOI:** 10.1186/s42826-026-00276-8

**Published:** 2026-04-07

**Authors:** A. Makris, N. Roshan, J. Iliopoulos, R. Waugh, A. Al-Hindawi, B. Corbett, C.C. Liu, B. Lian, S. Heffernan, J. Hyett, J. Ardui, S. Pears, S.A. Karumanchi, A. Hennessy

**Affiliations:** 1 South Western Sydney Local Health District, Sydney, Australia; 2 Western Sydney University, Sydney, Australia; 3 Ingham Institute for Applied Medical Research, 1 Campbell St, Liverpool, NSW Australia; 4 Sydney Local Health District, Sydney, Australia; 5 Kolling Institute of Medical Research, University of Sydney, St. Leonards, NSW Australia; 6 Cedars Sinai Medical Center, Los Angles, USA; 7 Faculty of Medicine and Health, University of Sydney, Sydney, Australia

**Keywords:** Preeclampsia, Baboons, Endovascular embolization, Surgical ligation, Uteroplacental ischaemia

## Abstract

**Background:**

Preeclampsia is a multisystem pregnancy disorder that is a major contributor to maternal and neonatal morbidity and mortality worldwide. An animal model facilitates further understanding of the disorder and allows for the investigation of targeted therapies prior to first in-human studies. Although there are several animal models for studying preeclampsia, the utero-placental ischemia (UPI) model is thought to best replicate the placental ischemia that develops as preeclampsia evolves in humans. An established non-human primate (NHP) model of UPI resembles human preeclampsia and has been important in understanding and finding cures for this disorder. To date the ischemia in most animal models has been undertaken by surgical ligation of a uterine artery. There are however alternate means to reduce arterial blood flow via endovascular means. This study aimed to investigate the difference between the surgical reduction in flow (surgical UPI) to the use of percutaneously delivered intra-arterial coils (coils UPI).

**Results:**

There was no significant difference between the two methods of inducing UPI for the measured parameters: circulating soluble fms-like tyrosine kinase-1(sFLT-1), blood pressure (BP) and proteinuria (*p* < 0.05). The results showed a percentage change from baseline in sFLT-1 of 3176 ± 1558% for the embolization and 2040 ± 1645% for the ligation group *p* = 0.11, three days post-UPI induction. Moreover, there was no difference between the two models in terms of preeclampsia-defining features.

**Conclusions:**

No statistically significant difference was found between the two methods, coils for endovascular embolization and surgical UPI. However, since endovascular embolization is a less invasive technique compared to ligation it may be preferred for induction of UPI in the NHP model.

**Supplementary Information:**

The online version contains supplementary material available at 10.1186/s42826-026-00276-8.

## Background

Preeclampsia is a leading cause of maternal and fetal morbidity and mortality with the major impact in low- and middle-income countries [1, 2]. The main clinical features are the new onset of hypertension after 20 weeks gestation in women, associated with the development of new end-organ dysfunction e.g. proteinuria [2]. The primary management strategy once preeclampsia develops is expectant management and ultimately delivery, which depending on the gestational age has the risk of preterm birth complications and adverse clinical outcomes [3, 4] and needs to be balanced against the risks of continuing the pregnancy. Novel therapies are evolving. Robust animal models are required to investigate therapeutic interventions, their mechanisms of action, and assess their safety and efficacy prior to testing in human clinical trials.

Many animal models have been developed to study preeclampsia e.g. genetic, inflammatory and immunological [5–12]. The UPI model is best thought to replicate the maternal clinical syndrome of preeclampsia. The UPI, in various species, has typically been established with total or incomplete surgical ligation of the uterine [13–16].

However, the invasive nature of this UPI model can be associated with inadvertent off-target tissue damage or ischemia (especially with aortic compression), inflammation, pain and recovery time due to the surgical procedure. An alternative method to reduce arterial blood flow is endovascular embolization. This can be achieved with the deployment of embolic agents intra-arterially (e.g. polyvinyl alcohol foam, microspheres, metallic coils or liquid polymers). These procedures are undertaken percutaneously and thus are less invasive and hence less painful from an animal welfare perspective [17], allowing for a more rapid recovery from the intervention.

Embolization is a common endovascular procedure which comparatively results in less post-operative mobility compared to open surgical procedures [17]. The use of coils in endovascular embolization has been an important fundamental intervention commonly used in human traumatic vascular injuries and arterial aneurysms [18].

Several studies have compared the outcomes of surgical versus endovascular treatment for aneurysms. Most of the human studies demonstrated a lower morbidity and mortality, lower rates of hospital discharge to long-term care [19]. In a retrospective study by Gorbaniet al., the two treatment options, endovascular coiling and microsurgical treatment were compared in management of ruptured middle cerebral artery (MCA) aneurysms in humans. A comparable result was reported showing the complete aneurysm occlusion of 90.5% and 89.5% by surgical clipping and endovascular coiling, respectively [20]. Another retrospective single centre study by Schwartz et al., assessed the long-term results of microsurgical clipping and endovascular coiling in treatment of MCA aneurysms. The result showed a higher complete occlusion rate of 96.3% clipping technique compared to endovascular therapy (78.9%); however, a better long-term clinical outcome was reported in the endovascular group [21].

Thus, the aim of this study was to compare in a non-human primate, the differences in the development of preeclampsia-like signs between an endovascular means of reducing uterine artery blood flow and surgical ligation.

## Methods

**Fig. 1:**
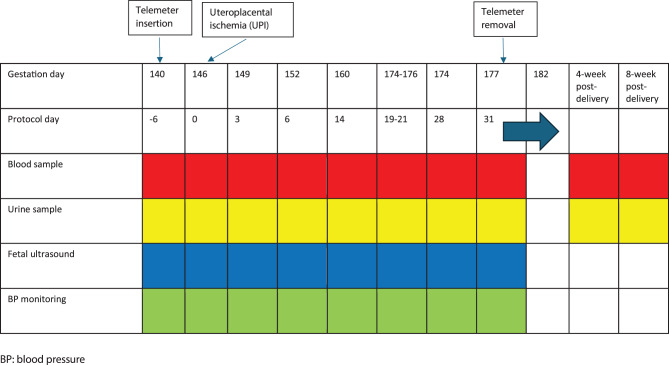
Timeline of the uteroplacental ischemia (UPI) procedure in a non-human primate model

Non-human primates (*Papio hamadryas*) at approximately 140 days of a 183-day long gestation, underwent UPI by either open uterine artery ligation (*n* = 9) or percutaneous endovascular coil deployment (*n* = 4). To adhere to the 3Rs principles of animal research (replacement, refinement, and reduction) [22] and avoid unethical repetition of control experiments, historical surgical ligation data were used as a comparison for the new technique with coils. To reduce bias as much as possible, all the experiments were undertaken by the same experimental team with essentially identical procedures except for the method of inducing UPI. Both groups were treated under similar conditions, including similar gestational age, standardized ketamine anesthesia, and adherence to institutional protocols approved by the Sydney Local Health District Animal Welfare Committee (protocols 2019/001 and 2023/003). Approximately one week prior to UPI, a subcutaneous telemeter was implanted for remote continuous intra-arterial BP measurement. Baseline blood pressure (BP) measurement, fetal ultrasound measures, as well as blood and urine were obtained approximately 48 hours prior to UPI, as described previously [15]. Blood, urine and fetal ultrasound assessments were performed pre- and post-UPI as outlined in Fig. 1. All procedures were undertaken under ketamine anesthesia (10 mg/kg, intramuscular injection) in both the surgical ligation (*n* = 9) and coil embolization (*n* = 4) groups. The dam and neonate were assessed 48 hours after delivery for overall welfare and wellbeing, sex, weight and length.

### BP measurement

The telemeters (Data Science Ltd™., Minnesota, USA) were implanted subcutaneously in the right or left flank. The catheter was tunnelled subcutaneously and inserted into the profunda femoral artery. The catheter is inserted such that the tip is in the aorta to the level of the renal arteries. BPs were measured continuously but assessed whilst animals were awake, defined as the period between 16:00 and 20:00. The BP during sleep was defined as the period between 23:00 and 03:00. BP data 5 mins before and 5 mins after an activity score of more than 300 (range: 0–4095 [23]) were removed to ensure BP was assessed at rest. Significant movement was defined as walking and more vigorous activity such as jumping onto a perch.

### Induction of UPI

Animals underwent either surgical uterine artery ligation, or endovascular uterine arterial embolization with the deployment of coils (Boston Scientific, Massachusetts, USA). The surgical ligation procedure was undertaken as described previously [15] and the animals included were historical controls. The uterine artery embolization procedure was undertaken on the non-dominant uterine artery. The artery was identified as non-dominant in supplying the placenta by using preoperative uterine arterial duplex (GE Healthcare, California, USA). The non-dominant artery was identified as the vessel with the higher resistance (as measured by the uterine artery pulsatility index). After palpating the femoral artery, the skin overlying was infiltrated with local anaesthetic (lignocaine hydrochloride, Pfizer, Australia). Under ultrasound guidance a micro-puncture kit was used to gain access to the artery and a 5 Fr Glide-sheath Slender® introducer sheath (Terumo Interventional Systems, New Jersey, USA) used to access the femoral artery for the procedure. Once the catheter was at the bifurcation of the aorta, an angiogram was undertaken to assess the total number of placental cotyledons supplied by both uterine arteries. A selective non- dominant uterine artery angiogram is undertaken to assess the number of cotyledons supplied by the unilateral artery. Once confirmed it’s the non- dominant side, coils are deployed to restrict totally the blood flow. A post procedure angiogram is undertaken to confirm the number of cotyledons remaining (Figure S1). In order to ensure there are no procedure related catheter-associated thrombosis, intravenous citrate (12.5 mg/min, Baxter Healthcare, Australia) was administered. Simultaneously, via a cannula in citrate (lateral arm intravenous calcium gluconate administered (31 mg/min, Phebra, NSW) to ensure normocalciuria. The procedure is undertaken and images captured using image intensification (Siemens Healthineers, Auckland, NZ).

### Specimen collection

All specimens at each time point were collected prior to the commencement of any surgical or endovascular procedures. One and a half mLs of blood was collected from the cephalic vein in the upper arm using aseptic technique [24]. The platelets, haemoglobin, creatinine, liver function tests, C-reactive protein and coagulation profile were measured. A further 5 mLs of blood was centrifuged, aliquoted and stored at −80 °C till analysis. Urine was collected by cannulating the urethra using a catheter at baseline (approximately 48 hours pre-UPI) and at designated post-UPI time points (as outlined in Fig. 1). Urinary spot protein (mg/L) and creatinine (mmol/L) were measured, and the ratio (mg/mmol) calculated. All testing was undertaken by a commercial NATA accredited laboratory.

### Enzyme-linked immunosorbent assay (ELISA)

A commercially available human enzyme-linked immunosorbent assay (R&D, Minneapolis, MN, USA) was used to measure the total soluble fms-like tyrosine kinase-1 (sFLT-1) in plasma. There have been changes made to the commercial assay over time and as such this data was presented as percentage change from baseline.

### Statistical analysis

Data were presented as mean±SD. Statistical analysis was performed (Prism 9) with mixed-effects analysis for repeated measures, (as multiple measurements were done in the same animal over time) and post-hoc Sidak test where applicable. Significance was set at *p* ≤ 0.05. Where comparisons between interventional groups were undertaken a Mann-Whitney U test was performed.

## Results

A total of 13 animals underwent UPI, 9 in the surgical group and 4 in the coils group. In both models placental blood flow was reduced by less than 50% of total flow as measured by cotyledon perfusion. was achieved.

### Blood pressure

**Fig. 2:**
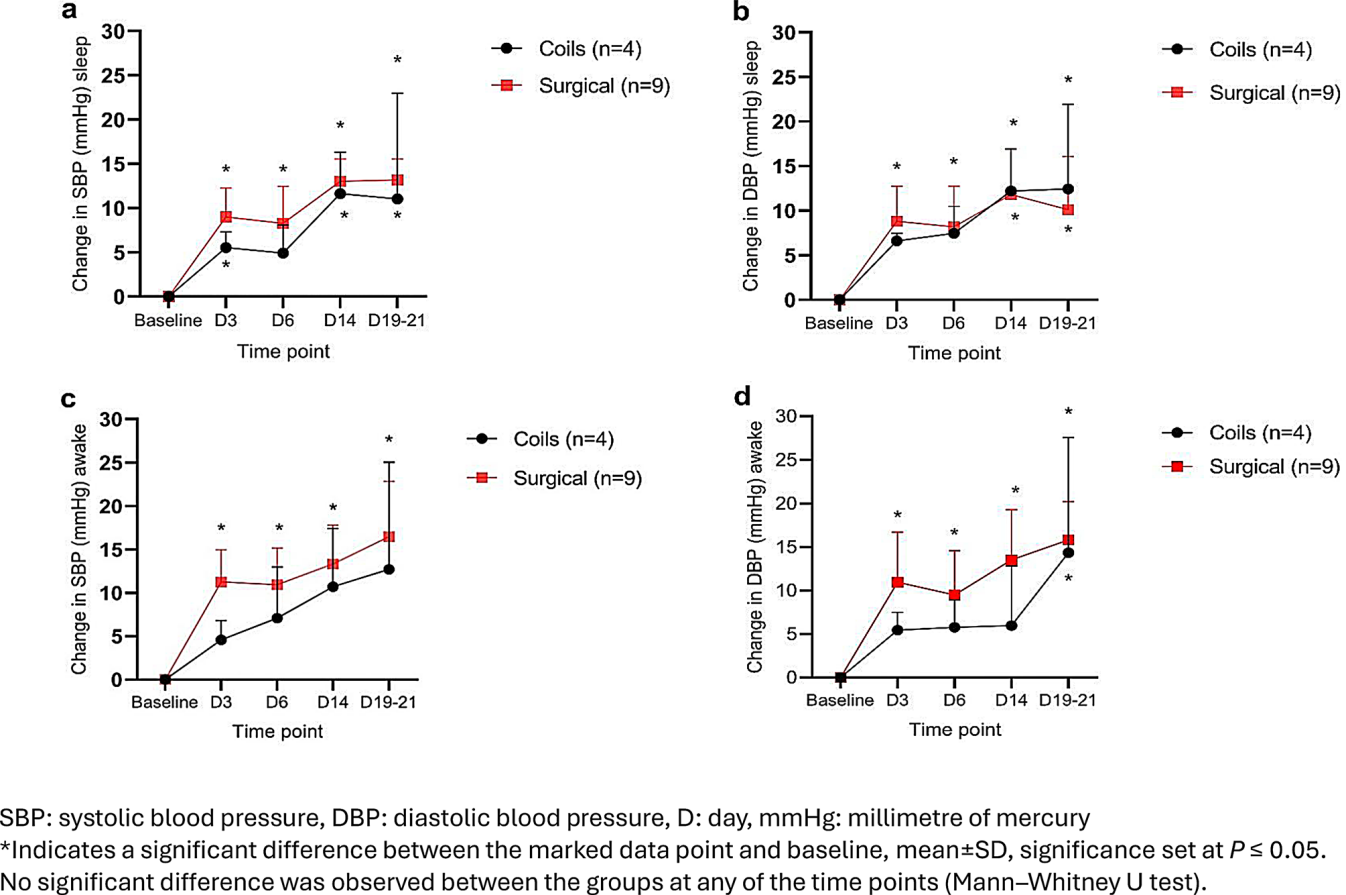
Systolic (SBP) and diastolic (DBP) blood pressure changes during sleep and wakefulness post-uteroplacental ischemia (UPI). The change in intra-arterial BP at sleep and awake periods post-UPI compared to baseline was monitored by an implanted telemeter in pregnant baboons undergoing either surgical ligation (*n* = 9, red squares) or endovascular arterial embolization (*n* = 4, black circles). **a**): sleep SBP change, **b**): sleep DBP change, **c**): awake SBP change, **d**): awake DBP change

The blood pressure increased in both models significantly as a result of the UPI compared to baseline measurements. There was no difference between the surgical and coil group with regards to changes in blood pressure over time, or during the awake or sleep periods. In the surgical group, the average elevation in systolic blood pressure (SBP) during the awake and sleep periods were 13.0 ± 4.7 and 10.8 ± 3.1 mmHg (both *p* < 0.001), and the elevation in diastolic blood pressure (DBP) at the awake and sleep periods were 12.5 ± 5.2 and 9.7 ± 4.9 mmHg, respectively (both *p* < 0.01) (Fig. 2).

In the coils group, there was a significant elevation in SBP during the sleep period (10.7±6.4 mmHg, *p* ≤ 0.01). There was also a significant elevation in DBP during the awake and sleep periods (14.4±13.2 mmHg and 14.3 ± 6.9 mmHg *p* ≤ 0.02; respectively) (Fig. 2).

### Proteinuria

**Fig. 3:**
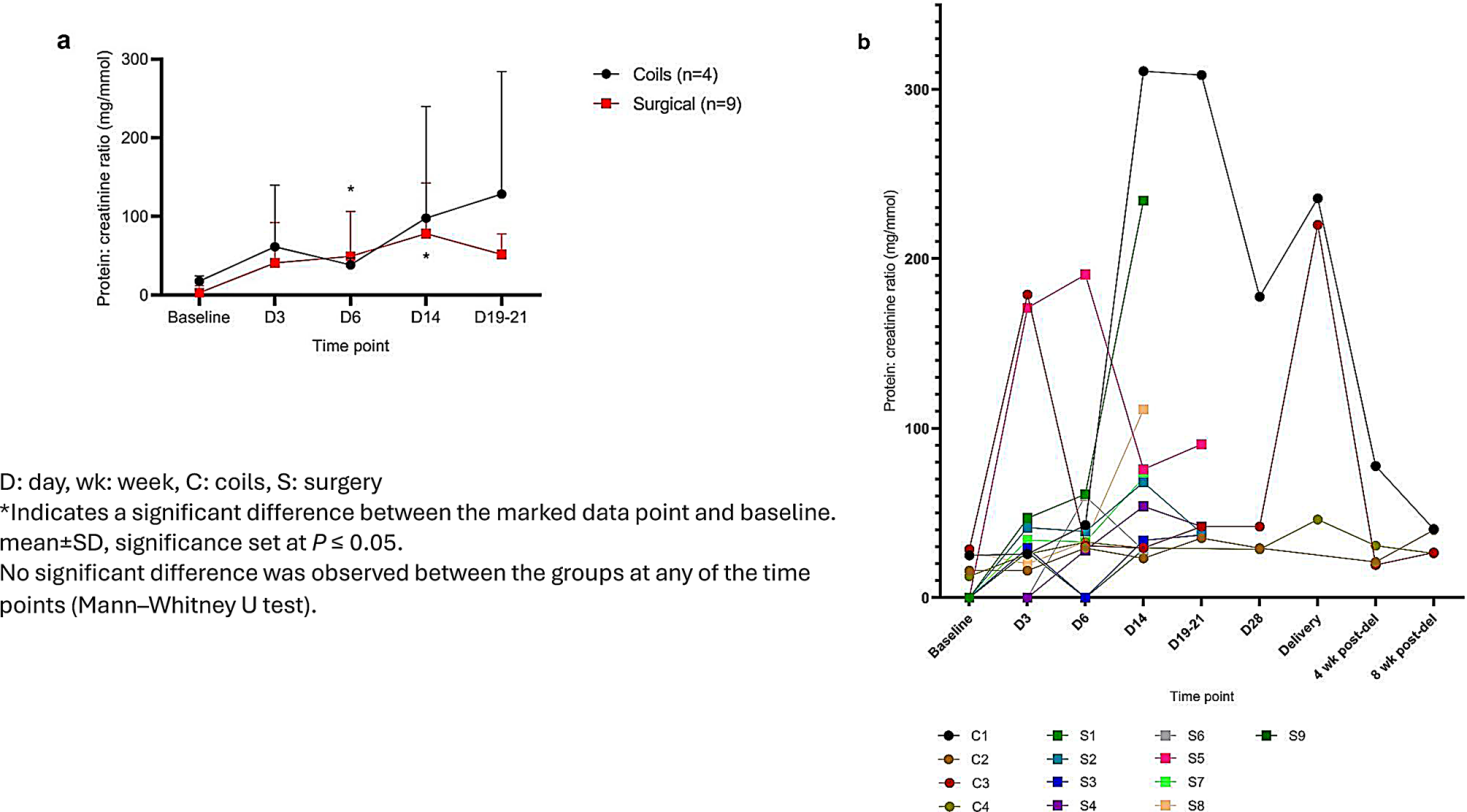
Urinary protein excretion pre- and post-uteroplacental ischemia (UPI). a): the excretion of urinary protein in pregnant baboons pre- and post-UPI at surgical ligation (*n* = 9, red squares) and endovascular arterial embolization models (*n* = 4, black circles). b): the urinary protein excretion presented case by case

The results for urinary protein excretion demonstrate there was no significant difference in proteinuria between the two models at any time points (Fig. 3a). Most of the animals in both models had an increase in urinary protein excretion post-UPI, but at different time points (Fig. 3b). Proteinuria was significantly increased compared to baseline in the coils group after 6 days of UPI with a protein: creatinine ratio of 40.0 ± 6.1 mg/mmol, and after 14 days in the surgical group (63.1±28.0 mg/mmol and both *p* < 0.05).

### Angiogenic biomarker

**Fig. 4:**
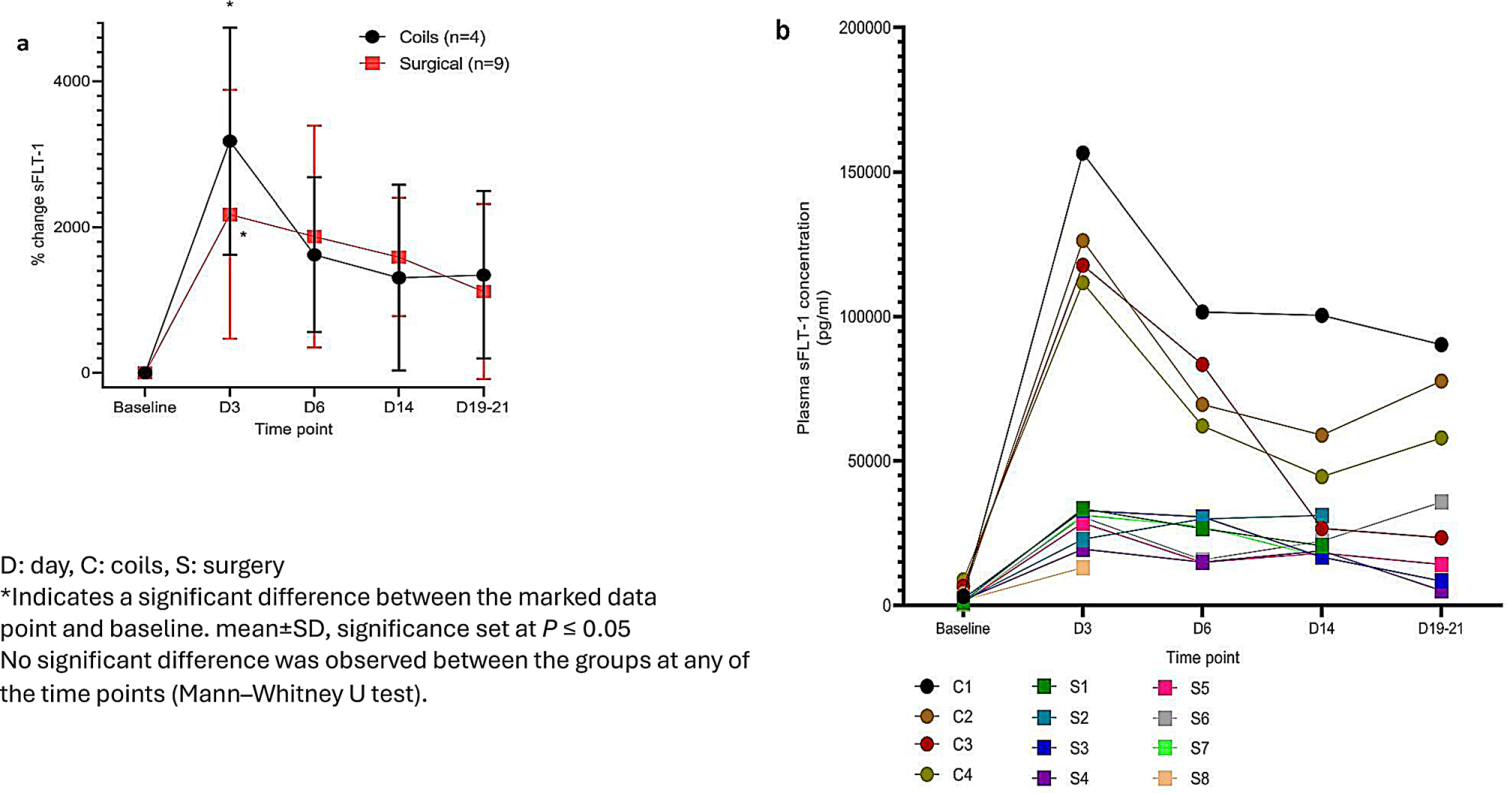
Change in plasma soluble fms-like tyrosine kinase 1 (sFLT-1) post-uteroplacental ischemia (UPI) compared to baseline. **a**): data presented as the % change in plasma sFLT-1 compared between the two models, surgical ligation (*n* = 9, red squares) and endovascular arterial embolization models (*n* = 4, black circles). **b**): data presented case by case

The circulating sFLT-1 (as a percentage change from baseline) was calculated due to the change in sensitivities of the commercially available ELISA as well as due to the significant baseline variation. There was no significant difference in sFLT-1 between the two models at any time points (Fig. 4a). A significant increase in plasma sFLT-1 concentration was evident soon after the UPI induction at day 3 (coils group: sFLT-1 3176 ± 1558% and surgical group: sFLT-1 2040 ± 1645%; *p* = 0.11 for comparison to baseline). Figure 4bdemonstrates the significant inter-animal variation the plasma sFLT-1 concentration.

### Other parameters

**Fig. 5:**
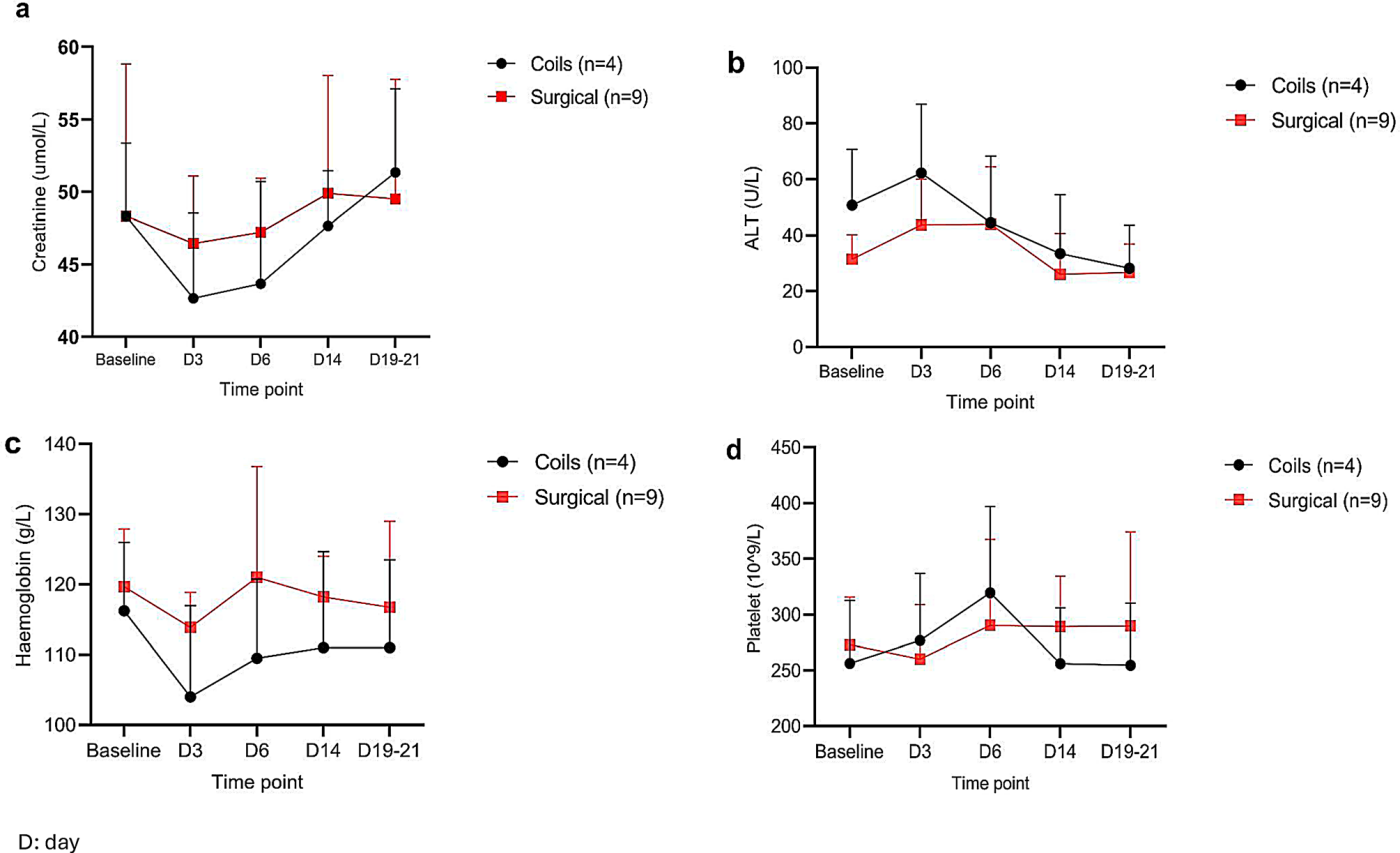
Other parameters measured pre- and post-uteroplacental ischemia (UPI). **a**): creatinine, **b**): alanine aminotransferase (ALT), **c**): haemoglobin, **d**): platelet count compared between the two models, surgical ligation (*n* = 9, red squares) and endovascular arterial embolization (coils) (*n* = 4, black circles), mean ± SD

The average creatinine, ALT, haemoglobin and platelet count in the coils group were 46.3 ± 3.9 µmol/L, 42.1 ± 15.0 U/L, 109 ± 3.3 g/L and 277 ± 30 respectively (Fig. 5). There was no significant difference (all *p* > 0.05) compared to surgical group (creatinine 48.2 ± 1.7 µmol/L, ALT 35.2 ± 10.1 U/L, haemoglobin 117 ± 3 g/L, and platelet count 282 ± 14 ×10^^9^/L). The CRP was not measured routinely at the time of the surgical UPI. However, in the animals undergoing UPI with coils, there was no significant change in the CRP during the protocol (baseline:1.5±0.7, post UPI: 2.5 ± 1.4; *p* = 0.5).

### Pregnancy outcome

There was no significant difference in the rate of live birth between the two models (*p* = 0.34). The surgical group had a 78% live birth rate (7 live birth out of 9 delivery), while the coils model showed a 100% live birth (4 live birth out of 4 deliveries). There was also no significant difference in the average baby weight (measured within 48hrs) between the two models (surgical group 0.74 ± 0.07 kg, coils group 0.72 ± 0.14 kg, *p* = 0.83).

## Discussion

Both models induced UPI and resulted in clinical measures and physiological changes akin to human preeclampsia without fetal growth restriction. Although the mechanism that was employed to induce the UPI was different, animals in both models experienced a similar increase in BP in most time points post-UPI, developed proteinuria and showed plasma biochemical changes noted in human. There was no significant difference between the two models in terms of measured parameters such as sFLT-1% change, BP, urinary protein excretion or proteinuria, birth outcomes, creatinine concentration, liver function tests, haemoglobin concentration and platelet count.

The increased concentration of antiangiogenic biomarkers such as sFLT-1 from the placenta is associated with clinical manifestations of preeclampsia. The sFLT-1 was first described by Kendall and Thomas in 1993 [25]. Although produced in the placenta and secreted from placental trophoblast cells into maternal circulation, the circulating sFLT-1 binds to vascular endothelial growth factor (VEGF) and placental growth factor (PlGF, a VEGF homology), inducing endothelial dysfunction. This leads to the clinical manifestations of preeclampsia by decreasing production of vasodilators such as nitric oxide, increased complement activation and endothelial expression of procoagulant [26]. The enhanced vascular activity and the disrupted nitric oxide signalling results in hypertensive phenotype in preeclampsia; it develops during pregnancy and subsides after delivery, implicating the placenta as the main pathogenic culprit. Moreover, the vascular dysfunction and microangiography may result in clinical expression of the disease in target organs such as kidney, liver and brain [27].

In this study, the change in BP was used as the end point to minimize the effect of inter-animal baseline variations. At all the time points post-UPI a trend of increase in BP was observed with the average being mostly > 5 mmHg compared to the baseline.

UPI resulted in an increase in urinary protein excretion in both models. The variation in proteinuria peak in the coil group (Day 6) compared to the surgical group (Day 14) may be due to the inter-animal differences, subtle differences in the degree of UPI or may indeed relate to the procedure. However, comparable proteinuria levels were shown in both groups, and all cases exhibited a similar trend. This variability in the presence, degree and timing of the development of proteinuria also occurs in women who develop preeclampsia [28].

Furthermore, considering the dichotomous assessment, the classical cut-off point that defines proteinuria in human pregnancy is a protein to creatinine ratio of >30 mg/mmol [29]. In both models the degree of protein excretion from day 6 to 19–21 of UPI was beyond the cut-off considered pathological in human, likely resulting from UPI-induced glomerular endotheliosis, with onset variations attributed to inter-animal differences in ischemia severity [15].

In animal models, the elevated concentrations of sFLT-1 correlated with reproduction of pathological manifestations of severe -features preeclampsia [15, 27]. In the both models, a positive relationship between the placental ischemia and increased sFLT-1 was observed. All the animals undergoing UPI followed a similar trend.

The NHP models particularly baboons are preferred for studying human preeclampsia, because their placental structure more closely reflects that of humans (i.e., is haemomonochorial), enabling proof of principle studies. Baboons also have singleton pregnancies and a closer molecular and genetic profile to human [30]. In contrast, in rodent models like the Reduced Uterine Perfusion Pressure (RUPP) model, the placenta structure, vascular physiology and immune system differ significantly from those of humans, and they have multiple offspring, which limit their translational relevance despite inducing preeclampsia-like features [30–32]. Both models enable therapeutic development, but the endovascular approach in the NHP model more accurately reflects human interventions [30–32].

Coils are amongst the most widely used embolic materials due to their relative ease of deployment and availability. Embolization using coils has been utilised for over 30 years; in recent years, their application has increased and, in some instances, replaced or reduced the need for open surgery in a variety of medical conditions, notable among which include the treatment of aneurysms [33].

In this study, endovascular coil embolization resulted in a model not different from surgical UPI in terms of preeclampsia-like outcomes, angiogenic markers and clinical parameters such as proteinuria and hypertension. Embolization with coils is less invasive for animals, has a faster recovery time, causes less inflammation, and allows for visualisation of the degree of UPI via digital subtraction angiography in the current protocol. The lack of change in the CRP is reassuring that there was no evidence of infection or significant tissue damage. The CRP has previously been identified to increase during periods of inflammation [34].

In the coils model, angiography was performed using radio-opaque contrast medium via intra-arterial injection resulting in the visualisation of blood flow in the left and right uterine arteries, helping to define the contribution of uterine artery to placental perfusion. In the ligation model, only ultrasound lateralisation was done, as such animals did not get exposed to a radio-opaque contrast and therefore the outcome not immediately visualised [35]. Notably, the radio-opaque contrast has no effect on glomerular proteinuria [36].

The clinical and biochemical effects of UPI demonstrated by both models in this project are applicable to human preeclampsia with elevated sFLT-1. This is a more targetable approach to treatment than was possible in the last when non-descript biomedical markers were used such as uric acid [37, 38].

The limitations of this study include the small sample size that was limited by high experimental cost, ethical considerations and length of animals’ gestation. Another limitation was the variation in time points of physiological changes due to individual animal responses. Future studies with larger sample size and mechanistic studies are needed to further validate these findings. In this study, historical controls were chosen as they were managed under similar conditions to the embolization group. All the experiments were undertaken by the same experimental team with essentially identical procedures except for the method of inducing UPI. This minimizes bias as much as possible and adheres to the 3Rs of animal research. While we took steps to minimize bias, we still recognize that concurrent studies could further diminish the potential bias but would be unethical to undertake repeat studies where comparator data is available [22]. Moreover, other potential confounding factors may include procedural events that happened at the time of the angiogram, for instance, the use of radio-opaque contrast and the administration of citrate and calcium gluconate. Although these confounders have a relatively short-lasting impact compared to the 21-day observation period of this study. Also, these events are intrinsic to the procedure undertaken. Similarly, the coils group did not have an open surgical procedure performed. These differences could not be controlled between groups.

## Conclusions

In conclusion, endovascular embolization with coils resulted in a model similar to surgical UPI in terms of preeclampsia-like outcomes in the development of a non- human primate model of preeclampsia. Endovascular embolization however is a less invasive technique with less preoperative requirements compared to ligation and may be preferred for induction of UPI in this NHP model. This technique allows the pre-clinical testing of treatment targeting the elevation in sFLT-1 associated with placental ischemia.

## Electronic supplementary material

Below is the link to the electronic supplementary material.

Supplementary material 1. Figure S1: Angiographic Images Demonstrating Changes Pre- and Post-Embolization. Images from digital subtraction angiography showing the non-dominant artery in a non-human primate (*Papio hamadryas*) before and after endovascular coil embolization to induce uteroplacental ischemia (UPI). a): Pre-embolization angiogram, showing normal blood flow in the uterine artery with clear visualisation of placental cotyledon perfusion. b): Post-embolization angiogram showing reduced cotyledon perfusion (a flow reduction to less than 50% of baseline), which confirms effective UPI induction. Images were captured using a Siemens Healthineers fluoroscopy system

## Data Availability

The datasets used and/or analyzed during the current study are available from the corresponding author on reasonable request with ethics approval.
