# Supplementary figures and images for: Endovascular coil embolization compared to surgical ligation of the uterine artery in a non-human primate model in a model of preeclampsia
Source: Lab Anim Res. 2026 Apr 7;42:12. doi: 10.1186/s42826-026-00276-8 (PMC13054988; doi:10.1186/s42826-026-00276-8)

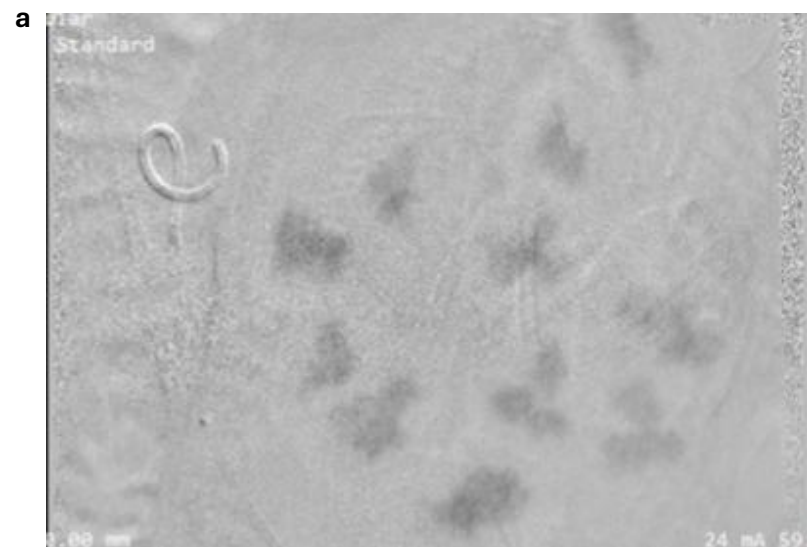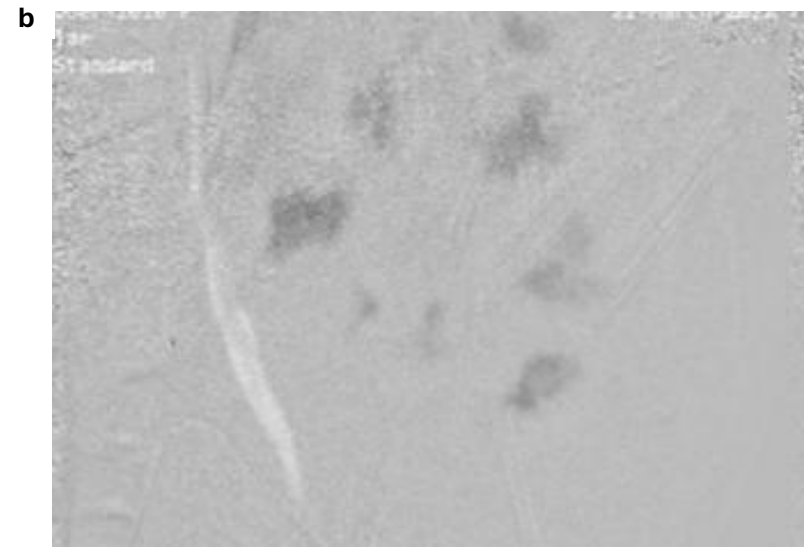

Supplement: Supplementary file 1 — Supplementary material 1. Figure S1: Angiographic Images Demonstrating Changes Pre- and Post-Embolization. Images from digital subtraction angiography showing the non-dominant artery in a non-human primate (Papio hamadryas) before and after endovascular coil embolization to induce uteroplacental ischemia (UPI). a): Pre-embolization angiogram, showing normal blood flow in the uterine artery with clear visualisation of placental cotyledon perfusion. b): Post-embolization angiogram showing reduced cotyledon perfusion (a flow reduction to less than 50% of baseline), which confirms effective UPI induction. Images were captured using a Siemens Healthineers fluoroscopy system [file 42826_2026_276_MOESM1_ESM.pdf]
